# Aripiprazole/Sertraline Combination: Clinical and Cost‐Effectiveness in Comparison With Quetiapine for the Treatment of Bipolar Depression (ASCEnD Trial)—Protocol for a Nested Qualitative Study

**DOI:** 10.1111/hex.70018

**Published:** 2024-09-04

**Authors:** Isobel Hoppe, Stuart Watson, Caroline Kemp, Fiona Turnbull, Firoza Davies, John Gibson, Lumbini Azim, Lauren Wall, Niraj Ahuja, Sarah Al‐Ashmori, Sally Keys, Thomas Kabir, Carolyn A. Chew‐Graham

**Affiliations:** ^1^ School of Medicine Keele University Newcastle UK; ^2^ Translational and Clinical Research Institute Newcastle University Newcastle upon Tyne UK; ^3^ Cumbria, Northumberland, Tyne and Wear NHS Trust Newcastle upon Tyne UK; ^4^ McPin Foundation London UK; ^5^ Cumbria, Northumberland, Tyne and Wear NHS Trust, Newcastle University Newcastle upon Tyne UK; ^6^ Regional Affective Disorders Service, Cumbria, Northumberland, Tyne and Wear NHS Trust Newcastle upon Tyne UK; ^7^ Clinical Trials Unit Newcastle University Newcastle upon Tyne UK; ^8^ Department of Psychiatry University of Oxford Oxford UK

**Keywords:** bipolar depression, bipolar disorder, clinical trial, mental health, nested qualitative study, patient and public involvement

## Abstract

**Introduction:**

Bipolar disorder is a recurrent mental health disorder with a prevalence rate of 1.4%. On average, there can be a delay of 9.5 years from the initial presentation of symptoms to a confirmed diagnosis. Individuals living with bipolar disorder have a reduced life expectancy. There is limited evidence regarding the effectiveness of antidepressants in treating bipolar disorder. The ASCEnD clinical trial will test the clinical and cost‐effectiveness of the aripiprazole/sertraline combination in comparison with quetiapine for the treatment of bipolar depression (individuals who suffer from depressive episodes in bipolar disorder) and will include a nested qualitative study.

**Methods:**

The qualitative study will use semi‐structured interviews to explore pilot trial participants' and clinicians' perspectives on recruitment procedures, the acceptability of the intervention, the management of bipolar disorder and attitudes to medication combinations.

**Conclusion:**

Findings will inform recruitment strategies and optimise training for the participating sites in the ASCEnD full trial. They will also help to illuminate the lived experience of people with bipolar disorder and the clinicians who work with people with bipolar disorder. The discussion will explore perspectives on the delay in diagnosis, having a diagnosis, the impact of living with bipolar disorder and attitudes to treatment, including drug combinations.

**Patient or Public Contribution:**

A Lived Experience Advisory Panel (LEAP) has been convened with the support of the McPin Foundation, which will contribute to the ASCEnD trial and its nested qualitative study to provide input on the design and delivery of the trial and qualitative study, analysis of qualitative data and dissemination of findings.

## 1 Introduction

Bipolar disorder is diagnosed on the basis of a current or previous episode of mania or hypomania [1, 2]. It is usually a lifelong relapsing and recurring illness [3, 4] with a lifetime prevalence rate of at least 1.4% [4, 5], meaning that 1.3 million people currently live with bipolar disorder in the United Kingdom [6] and an estimated 40 million worldwide [7]. The disorder is associated with a reduced life expectancy of 8–12 years [8] due to several reasons, including comorbid physical health problems (e.g., cardiometabolic diseases) caused by antipsychotics and suicide [9, 10, 11]. In the United Kingdom, bipolar disorder has an estimated treatment cost of £5.2 billion annually, with direct NHS costs of £342 million [12, 13]. This figure is placed at $202 billion in the United States [14].

Furthermore, evidence suggests that there is an average delay of 9.5 years between the early manifestations of bipolar disorder and its diagnosis/treatment [15]. People with bipolar disorder are usually symptomatic about half of the time [16, 17, 18, 19], with depression appearing to be the most prevalent symptom, and 50% of patients remain depressed for up to 6 months at a time (‘bipolar depression’). Nevertheless, depression responds differently in people living with bipolar disorder compared to unipolar depression, with limited evidence for the effectiveness of pharmacological and psychological interventions for bipolar depression [20]. Along with psychological interventions (e.g., cognitive behavioural therapy), the National Institute for Health and Care Excellence (NICE) guideline (CG185) currently recommends quetiapine, olanzapine (with or without fluoxetine) and lamotrigine for those with bipolar depression [21]. These guidelines are echoed internationally, with most guidelines variably recommending mood stabilisers and antipsychotics [22]. This contrasts with a range of antidepressants recommended to those with a major depressive disorder (unipolar depression) [23]. There is clinical uncertainty around the use of antidepressants and antipsychotics in bipolar depression, an area that both NICE and the James Lind Alliance (JLA) agree requires more research [21, 24].

The ASCEnD trial will test the clinical and cost‐effectiveness of the aripiprazole/sertraline combination in comparison with quetiapine for the treatment of bipolar depression [25]. The trial will be conducted at 10–11 UK sites, including primary, secondary and tertiary care mental health services. A target of 270 patients will be randomised in a 1:1 ratio to receive either a sertraline/aripiprazole combination or quetiapine. The effectiveness of either a sertraline/aripiprazole combination or quetiapine in reducing depressive symptoms (the primary outcome measure) will be assessed 12–16 weeks after randomisation. The protocol for the main trial will be published separately in due course.

The National Institute for Health and Care Research (NIHR) stresses that patients and the public should be active partners in all aspects of NHS public health and social care research, including design and delivery [26]. Research should be ‘carried out with or by members of the public rather than to, or about, or for them’ [27]. Despite this, research on bipolar disorder that includes patient and public involvement (PPI) is limited [28, 29]. The benefits of PPI in research can include the identification of outcomes that are valued by patients, user‐friendly research design, appropriate recruitment strategies, coherent and comprehensible data that are interpreted correctly and enhanced quality of research outputs with widespread dissemination of findings [30, 31, 32]. Involvement can take place at different levels of effort, impact, commitment and outcome [33]. Ideally, patients and the public should be active participants in decision‐making and co‐production in all stages of the research, thereby helping the research team to increase the pool of knowledge, skills and experiences.

In recognition of the value of PPI, a Lived Experience Advisory Panel (LEAP) has been convened with the support of the McPin Foundation. The panel consists of 10 members recruited to ensure diversity in sex, age, ethnicity and background. The LEAP will contribute to the ASCEnD trial and its nested qualitative study to provide input throughout the trial design and delivery, as well as the dissemination of findings.

This article focuses on a nested qualitative study conducted within the internal pilot of the clinical trial, set to run for 9 months from the start of the recruitment period, with the aim of informing recruitment strategies and optimising training for the participating sites. Current literature suggests that there is an exceptionally high dropout and non‐adherence rate in patients living with bipolar disorder in clinical trials, particularly amongst recently diagnosed patients [34, 35, 36]. Therefore, this nested study aims to explore the reason for this by examining the experiences of the ASCEnD clinical trial's recruitment procedures, the acceptability of the intervention (aripiprazole/sertraline combination in comparison with quetiapine) and perspectives and challenges in the diagnosis and management of bipolar disorder. To do so, we will interview trial participants and clinicians, including GPs and psychiatrists [37].

## 2 Materials and Methods

NHS Research Ethics Committee (REC) approval has been granted for the project (Reference Number 23/NE/0132).

### 2.1 Design

A qualitative study using semi‐structured interviews with 25 trial participants will be conducted to explore the acceptability of the recruitment procedures, trial drugs, perspectives on the diagnosis and management of bipolar disorder and attitudes to medication and medication combinations. We will also conduct interviews with 30 clinicians involved in the ASCEnD clinical trial to explore the acceptability of the recruitment procedures and trial drugs and examine current difficulties in diagnosing and managing bipolar disorder, challenges accessing/delivering specialist opinions within current NHS systems and change in workload, particularly with the forthcoming community mental health transformation framework [37].

The findings will inform recruitment strategies and optimise training for trial sites in the full trial (beginning in month 10 following the pilot trial), help inform potential future implementation of the use of the quetiapine and aripiprazole/sertraline combination into clinical practice and provide an understanding of the lived experience of people with bipolar disorder and clinicians supporting them.

A LEAP group has been convened, which will contribute to the ASCEnD clinical trial and its nested qualitative study. LEAP members and study co‐investigators (J.G. and T.K.) have already contributed to the development of public‐facing documents submitted for ethics approval (including protocol), helped to develop topic guides and training videos, contributed to the ASCEnD website structure and content and joined trial management group (TMG) meetings. In the future, the LEAP will continue to contribute to the modification of topic guides, public‐facing documents, data analysis and dissemination activities and share their thoughts at LEAP and TMG meetings.

### 2.2 Participant Sampling and Recruitment

#### 2.2.1 ASCEnD Trial Participants

Fifteen participants who complete the pilot trial and have given their consent to be contacted will be invited to participate in a single semi‐structured interview after the primary outcome data have been collected (Weeks 12–16) to explore experiences of the recruitment procedures, acceptability of the intervention and their experience of diagnosis and management of bipolar disorder. They will be identified by the lead trial site and passed to the qualitative researcher (I.H.). Participants' contact details will be stored securely at Keele University.

Ten participants who withdrew from the pilot trial but agreed to be contacted to participate in an interview (as per the informed consent form) will be invited to take part in a single interview to explore reasons for withdrawal, including trial processes or medication concerns, and their experience of diagnosis and management of bipolar disorder. We will aim to sample people with a range of genders, ages and ethnicities (both those with recent and long‐standing diagnoses). Trial participants will be asked if they wish to have a friend/carer to support them during the interview. All participants will be reimbursed with a £25 shopping voucher for their time.

#### 2.2.2 Clinicians

Purposive sampling will be used to identify 15 GPs and 15 psychiatrists involved in the pilot trial, who will be invited to participate in an interview to explore the acceptability of the recruitment procedures and the trial drugs, as well as their experience of diagnosis and management of bipolar disorder. We will aim to sample from a range of trial sites and individual genders, ages and ethnicities. NHS trusts involved in the clinical trial will be contacted to discuss recruitment options for the nested study between May 2024 and December 2024.

Staff who are involved in the study will be documented in the site's study delegation log. The lead trial management team will provide the names of the staff involved in the trial to the researchers at Keele University, along with their contact details (emails) and their principal investigator's contact details. Staff will also be recruited through NHS trusts that have Patient Identification Centres (PICs), professional networks, social media and snowball sampling. Because GPs are self‐employed, they will be reimbursed for their time according to British Medical Association (BMA) rates (£88/h).

### 2.3 Data Collection

Semi‐structured interviews will be conducted by the qualitative researcher (I.H.) according to participant preference, either by telephone, using an online platform or face‐to‐face (at a place and time convenient to them, e.g., practice address). The interviewer will take a constructivist approach while striving to ensure that they remain engaged and open to the development of new ideas and themes. The interviewer will seek to respect varying views and ideas from each of the interviewee's independent social, cultural, political and ideological perspectives.

Interviews will be guided by topic guides, which will include open‐ended questions and prompts. Topic guides will be developed with reference to the existing literature and with input from the LEAP and co‐investigators T.K. and J.G.

Topic guides for trial participant interviews will explore the reasons for participating in the ASCEnD trial and continuing or withdrawing to inform recruitment strategies for the full trial. Topic guides will also allow for an exploration of experiences of mental health problems and receiving the diagnosis of bipolar disorder, experiences with different sorts of medication and views on drug combinations.

The topic guides for GPs and psychiatrists will explore their experiences of diagnosing and managing bipolar disorder, accessing or delivering specialist opinion within current NHS systems and participating in the ASCEnD trial.

Each interview is expected to last up to 45 min. The topic guides will be modified iteratively as data collection and analysis occur, with input from the LEAP to facilitate further exploration of key themes. Interviews will be digitally recorded with consent.

We aim to recruit up to 15 participants who completed the trial (whether or not they changed their medication) and approximately 10 people who consented to participate in the trial and withdrew (but consented to be interviewed). We aim to interview up to 15 GPs and 15 psychiatrists. It is anticipated that this number of participants will be sufficient to achieve data saturation in each data set [38].

### 2.4 Data Analysis

Digitally recorded interviews will be transcribed verbatim by an external transcription company with which we hold a confidentiality agreement. Each transcript will be anonymised by the qualitative researcher and given a unique participant identifier number. Transcriptions will be checked for accuracy by the interviewer before being analysed by Glaser and Strauss's method of constant comparison [39]. An inductive approach using thematic analysis [40] will be conducted, looking for connections within and across interviews and across codes, highlighting data consistencies and variations. A sample of initial transcripts will be independently coded by members of the qualitative team to develop categories and themes to be discussed at trial management and LEAP meetings. The analysis will be an iterative process, carried out in collaboration with the study LEAP, with emergent findings used to further refine topic guides for subsequent interviews. The thematic analysis within data sets will be followed by a framework analysis based on the theoretical framework of acceptability [41] across the data sets to inform recruitment strategies for the full trial.

The qualitative study findings will be discussed in LEAP and TMG meetings to inform the refinement of recruitment procedures and other aspects of the full trial.

### 2.5 Ethics and Data Management Plan

All participants will be given an information sheet about the study before the interview. Participants will be guaranteed anonymity and will be made aware of their right to withdraw at any point before data analysis has been conducted for any reason at any time or not answer any question they choose not to. If participants choose to withdraw prior to any transcription, their data collected in the interview will be destroyed. Before the interview, participants will be sent a patient information sheet that includes sufficient information about the study. Verbal consent will be obtained at the beginning of each interview.

Sensitive and personal data will be held and managed in line with the conditions of the study's ethical approval from the Health Research Authority, the UK Policy for Health and Social Care Research, the Data Protection Act and Keele University policies. Anonymisation of electronic sensitive data will be undertaken. Personal data will only be accessible to the research team during the data collection phase of the study. A study database containing participant information will be stored on Keele University's secure network in a password‐protected folder in the OneDrive storage area of the project. Personal data and digital recordings will be kept for up to 6 months after the end of the qualitative study; this will be password‐protected and stored in a different file from the transcripts. The transcripts will be kept for 5 years after the end of the qualitative study. Research data will be pseudo‐anonymised before analysis using a unique study code; only members of the study team based at Keele University will have access to the OneDrive folder to identify data, as Keele University is the data controller for this study. Pseudo‐anonymised data will be shared securely via email with the lead university as required. Pseudo‐anonymised data will also be shared with the TMG and LEAP members for feedback.

## 3 Discussion

The nested qualitative study within the ASCEnD pilot trial will inform recruitment strategies and optimise training for the full trial. In addition, interviews with people living with bipolar disorder will add to the limited understanding of their lived experiences of delayed diagnosis and management offered by the National Health Service; interviews with clinicians will illuminate reasons for delay in diagnosis, views of medication regimens and communication across the primary/secondary care interface.

Treatment‐resistant mood disorders are one of the leading challenges for clinicians today; hence, exploring patient perspectives on interventions and medication regimens is crucial. Concordance to some medications, particularly antipsychotics used in the management of people with mental health problems, is low. One estimate is that ‘31.0% were non‐adherent to ≥ 20% of their mood stabiliser and/or antipsychotic prescriptions’ [42]. There are several reasons for poor concordance, including unpleasant side effects such as weight gain, tremors and cardiovascular diseases [43]. Additional clinical and economic reasons include the need to take time off work, excessive hospital visits and negative impacts on relationships with friends and family [44].

Therefore, it is essential to look at not just the efficacy of intervention and medication regimens but also their acceptability from a patient perspective, considering a comprehensive view of both the beneficial and unwanted effects of interventions and the medication(s) being studied. Similarly, it is vital to understand the perspectives of healthcare professionals on interventions and complex medication regimens because this influences patient management and prescribing behaviour. Qualitative studies provide a way of doing this in a comprehensive and sensitive way. By using qualitative research, we hope to unveil findings that cannot be found in quantitative work by constructing a narrative for those involved in the ASCEnD trial.

Healthcare professionals, research staff and members of the LEAP have, and will continue to, work together on trial design, delivery, analysis and interpretation of data and dissemination of findings, including the development and modification of topic guides, public‐facing documents, data analysis and dissemination activities to deliver a successful nested qualitative study and clinical trial.

Findings will be shared with the wider TMG at monthly meetings to inform the refinement of recruitment procedures and optimise training at each of the NHS trial sites. Findings related to the experiences of diagnosis and management of bipolar disorder will be disseminated in journals to academic audiences and a lay audience in partnership with the McPin Foundation and Bipolar UK. We will use the COREQ checklist when reporting our findings [45]. We will also develop blogs and an infographic to share findings with lay and clinical audiences. Findings will also be shared with clinical audiences at conferences such as the Royal College of General Practitioners and the Royal College of Psychiatrists.

## 4 Strengths and Limitations

This nested qualitative study will inform recruitment strategies and optimise training for the principal ASCEnD randomised controlled trial. In addition, the findings will contribute to knowledge about the lived experience of bipolar disorder, including perspectives on delay in diagnosis and living with and impact of bipolar disorder [10]. Finally, insights may also help to better understand the motivations and experiences of people living with bipolar disorder, engaging in research.

Nevertheless, this study does not come without its limitations. Although we intend to invite ASCEnD trial participants who withdraw from the trial to be interviewed, such people may not be willing to participate in an interview, so this perspective (which is vital to inform trial design and recruitment) may be under‐represented. Furthermore, although we have diversity in our LEAP members in terms of gender, age and ethnicity, we do not have geographically diverse participants, with eight out of 10 members from the North‐East of England. However, this allows for regular in‐person LEAP meetings.

## Author Contributions

**Isobel Hoppe:** writing–original draft, methodology, investigation, writing–review and editing, visualisation. **Stuart Watson:** conceptualisation, supervision, funding acquisition, validation, writing–review and editing. **Caroline Kemp:** validation, writing–review and editing. **Fiona Turnbull:** validation, writing–review and editing. **Firoza Davies:** validation, writing–review and editing. **John Gibson:** validation, writing–review and editing. **Lumbini Azim:** data curation, investigation, writing–review and editing. **Lauren Wall:** data curation, investigation, writing–review and editing. **Niraj Ahuja:** writing–review and editing, visualisation, supervision. **Sarah Al‐Ashmori:** formal analysis, writing–review and editing. **Sally Keys:** validation, writing–review and editing. **Thomas Kabir:** validation, writing–review and editing. **Carolyn A Chew‐Graham:** conceptualisation, supervision, writing–review and editing, funding acquisition, validation.

## Conflicts of Interest

Carolyn A. Chew‐Graham is editor in chief of Health Expectations. The other authors declare no conflicts of interest.

## Data Availability

Data sharing is not applicable to this article as no data sets were generated or analysed during the current study.
